# Analysis of Programmed Cell Death and Senescence Markers in the Developing Retina of an Altricial Bird Species

**DOI:** 10.3390/cells10030504

**Published:** 2021-02-26

**Authors:** Guadalupe Álvarez-Hernán, José Antonio de Mera-Rodríguez, Ismael Hernández-Núñez, Alfonso Marzal, Yolanda Gañán, Gervasio Martín-Partido, Joaquín Rodríguez-León, Javier Francisco-Morcillo

**Affiliations:** 1 Área de Biología Celular Departamento de Anatomía Biología Celular y Zoología, Facultad de Ciencias, Universidad de Extremadura, 06006 Badajoz, Spain; galvarezt@unex.es (G.Á.-H.); merarodja@unex.es (J.A.d.M.-R.); ihernandezn7694@gmail.com (I.H.-N.); gmartin@unex.es (G.M.-P.); 2 Área de Zoología, Departamento de Anatomía, Biología Celular y Zoología, Facultad de Ciencias, Universidad de Extremadura, 06006 Badajoz, Spain; amarzal@unex.es; 3 Área de Anatomía y Embriología Humana, Departamento de Anatomía, Biología Celular y Zoología, Facultad de Medicina, Universidad de Extremadura, 06006 Badajoz, Spain; yolandag@unex.es

**Keywords:** programmed cell death, cellular senescence, retinogenesis, altricial bird species, precocial bird species, senescence-associated galactosidase activity

## Abstract

This study shows the distribution patterns of apoptotic cells and biomarkers of cellular senescence during the ontogeny of the retina in the zebra finch (*T. guttata*). Neurogenesis in this altricial bird species is intense in the retina at perinatal and post-hatching stages, as opposed to precocial bird species in which retinogenesis occurs entirely during the embryonic period. Various phases of programmed cell death (PCD) were distinguishable in the *T. guttata* visual system. These included areas of PCD in the central region of the neuroretina at the stages of optic cup morphogenesis, and in the sub-optic necrotic centers (St15–St20). A small focus of early neural PCD was detected in the neuroblastic layer, dorsal to the optic nerve head, coinciding with the appearance of the first differentiated neuroblasts (St24–St25). There were sparse pyknotic bodies in the non-laminated retina between St26 and St37. An intense wave of neurotrophic PCD was detected in the laminated retina between St42 and P8, the last post-hatching stage included in the present study. PCD was absent from the photoreceptor layer. Phagocytic activity was also detected in Müller cells during the wave of neurotrophic PCD. With regard to the chronotopographical staining patterns of senescence biomarkers, there was strong parallelism between the SA-*β*-GAL signal and p21 immunoreactivity in both the undifferentiated and the laminated retina, coinciding in the cell body of differentiated neurons. In contrast, no correlation was found between SA-*β*-GAL activity and the distribution of TUNEL-positive cells in the developing tissue.

## 1. Introduction

Programmed cell death (PCD) and cellular senescence during vertebrate embryogenesis are transient phenomena that contribute mainly to tissue remodeling [1,2,3] through the degeneration of temporary structures in the embryo. Indeed, it has been described that PCD processes are accompanied by cell senescence in interdigital regression [4,5,6], heart morphogenesis [7], pronephros and mesonephros degeneration [8,9,10,11], and degeneration of structures in the developing otic vesicle [12,13,14].

The vertebrate visual system constitutes an excellent model for investigating the mechanisms involved in cell degeneration and the phases of PCD that affect different structures (for a review, see [3]). Areas of intense PCD have been described in the developing visual system in fish [15,16,17,18,19], amphibians [20,21,22], reptiles [23,24,25], and mammals [26,27,28,29,30,31]. With respect to birds, similar studies have been conducted in the chicken [32,33,34,35,36,37,38] and in the quail [39], two precocial bird species. In these species, PCD during visual system morphogenesis and retinogenesis is completely restricted to the embryonic period. During early stages of avian eye morphogenesis, two pyknotic zones have been described in the central region of the retinal neuroepithelium and in the dorsal rim of the optic cup [35]. Furthermore, two areas of intense PCD appear in the neuroepithelium located laterally to the optic chiasm, in the so-called sub-optic necrotic centers (SONCs) [40,41]. With the onset of neurogenesis in the neural retina, PCD also affects neuroepithelial cells and newborn ganglion cell neuroblasts [33,36,37,38]. At later stages, coinciding with the synaptogenesis between retinal neurons, PCD affects those neurons that are unable to successfully innervate their targets [34,37,39]. The last wave of cell death follows different gradients that resemble the spatiotemporal patterns of cell differentiation [34,39].

With regard to developmental cellular senescence, several markers are currently employed to identify the distribution of senescent cells in vertebrate embryos. One of the most commonly used is the histochemical technique that detects the presence of β-galactosidase enzymatic activity at pH 6.0 (senescence-associated β-galactosidase, SA-β-GAL), different from that normally observed at pH 4.0 within lysosomes [42]. Increased expression of intracellular proteins such as p21, p16, p63, and p73 and the Btg/Tob tumor suppressor gene family also identifies cell senescence in several regions of the developing embryo [6]. These markers have been described in different embryonic tissues, but little is known about their distribution in the developing visual system. In this sense, we have recently described that some of these senescence markers are detected not only in several subpopulations of neurons in the developing retina, but also in the retinal pigment epithelium [43,44].

Although the ontogenetic mechanisms involved in visual system development and the basic structure of the retina are similar across bird species, the developmental rate and the acquisition of retinal structures are highly variable. Visual system morphogenesis and retinogenesis occur early in embryogenesis in precocial bird species [45,46], while these ontogenetic processes are delayed in altricial birds [47,48,49]. This delay can reach the stage of hatching and the first week of life, in which intense postnatal neurogenesis has been detected in the altricial retina [50]. The timing of histogenesis and cell differentiation and the state of retinal maturation at hatching thus differ significantly between precocial and altricial bird species.

All these data suggest that it is necessary to study visual system development across a broad range of avian species to conduct interspecific comparisons that can clarify the ontogenetic patterns. In the present study, we use classical histological, histochemical, and immunohistochemical methods (i) to describe the chronotopographical patterns of cell death and cell senescence markers in the developing visual system of an altricial bird species, the zebra finch (*Taeniopygia guttata*, Vieillot 1817), (ii) to study whether the distribution of senescence markers correlates with the progression of cell death in the *Taeniopygia guttata* retinal tissue, and (iii) to compare these results with those described in other precocial bird species, such as *Gallus gallus* or *Coturnix japonica*, and in the rest of the vertebrates.

## 2. Materials and Methods

### 2.1. Animal and Tissue Processing

**Figure 1:**
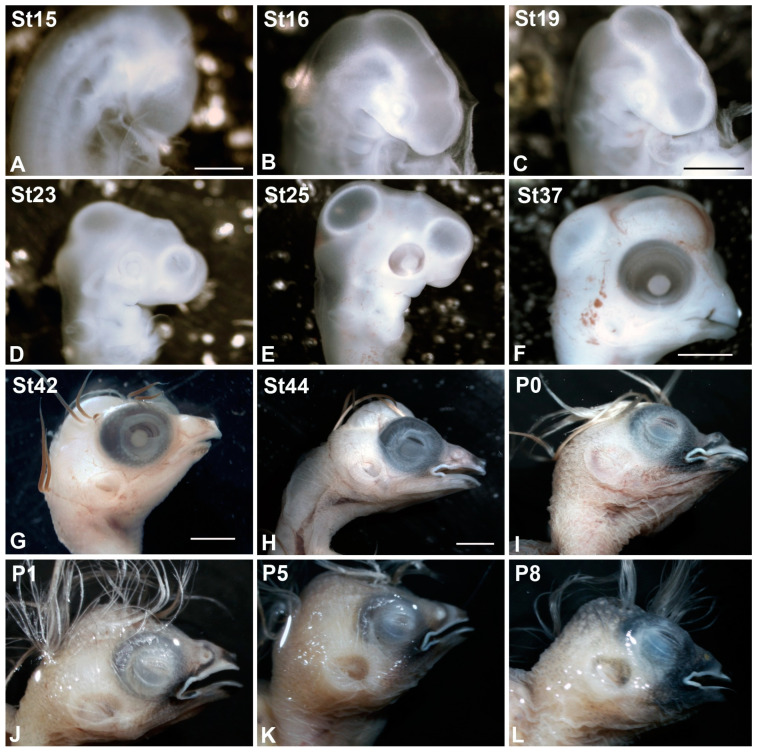
Stereomicroscope images of some embryos and postnatal specimens of *Taeniopygia guttata* showing the external morphological changes of the eye. The embryos were staged in accordance with the developmental stages (St) established by [51]. The optic cup was distinguishable between St15 and St23 (**A**–**D**). Pigmentation in the RPE was observed at St25 (**E**). At St37, the eye was completely pigmented (**F**). From St42 until perinatal stages, the eyelids progressively covered the eye (**G**–**J**). Eyelids were closed at P5 (**K**), but slightly open at P8 (**L**). Scale bars: 2 mm (**A**,**B**); 3 mm (**C**–**E**); 6 mm (**F**); 7 mm (**G**); 10 mm (**H**–**L**).

All animals were treated according to the regulations and laws of the European Union (EU Directive 2010/63/EU) and Spain (Royal Decree 53/2013). A total of twenty-seven *T. guttata* embryos and twelve hatchlings were used in the present study (Table 1). Embryos were obtained by incubating eggs in a rotating egg incubator (Masallés S.A., Spain) that was maintained at 37.5 ± 1 °C, 80–90% humidity. The degree of development of the embryos and hatchlings (Figure 1) was determined in accordance with the stages (St) established by by [51]. Embryos and hatchlings were fixed with paraformaldehyde (PFA) 4% in phosphate-buffered solution (PBS) (0.1 M, pH 7.4) overnight at 4 °C. For histological analysis with toluidine blue staining, some fixed embryos were dehydrated in a graded series of acetone and propylene oxide and embedded in Spurr’s resin. Serial frontal 3 µm sections were cut in a Reichert Jung microtome.

For the histochemical and immunohistochemical procedures, embryos and hatchlings were immersed overnight in a cryoprotective solution (15% sucrose in PBS) at 4 °C, soaked in embedding medium, and frozen. Cryosections of 20 µm were obtained in a cryostat microtome (Leica CM 1900, Charleston, SC, USA), thaw mounted on SuperFrost Plus slides, air dried, and stored at 20 °C.

### 2.2. Toluidine Blue Staining

Morphological analysis of development of cell death was conducted on resin sections stained with toluidine blue 0.5% and sodium tetraborate 0.5% solution. For this purpose, slides were put in the colorant at 90 °C for 45 s and then rinsed with distilled water. Sections were mounted with Eukitt (Kindler, Freiburg, Germany).

### 2.3. Detection of β-Galactosidase Activity

We followed the protocol described by [52]. Cryosections were incubated in 450 µL of chromogenic SA-*β*-GAL substrate X-gal (5-bromo-4-chloro-3-indolyl-*β*-d-galactopyranoside) in *PBS*−*MgCl*2 at pH 6.0 at 37.5 °C for 24 h. A blue-green precipitate was developed by *SA−β− GAL*-positive cells. Then, sections were washed in *PBS* −*MgCl*2 acid buffer for 10 min. After histochemical reaction, some of the sections were counterstained with DAPI (Sigma-Aldrich, Madrid, Spain, Ref. D9542) and others were used to perform immunohistochemical analyses. Slides were rinsed in PBS and mounted with Mowiol (Polyvinyl alcohol 40–88, Fluka, Madrid, Spain, Ref. 81386).

### 2.4. Immunohistochemistry

After histochemical analyses to detect *β*-galactosidase activity, slides were subjected to an antigen retrieval process with citrate buffer (pH 6) at 90 °C or 30 min. Sections were chilled at RT for 20 min. Slides were washed several times in 0.1% Triton-X-100 in PBS (PBS-T) and pre-blocked in 0.2% gelatin, 0.25% Triton-X-100, and Lys 0.1M in PBS (PBS-G-T-L) for 1 h.

Sections were incubated with mouse anti-p21 monoclonal antibody (1:200, Abcam, Madrid, Spain, ab109199) overnight at RT in a humidified chamber. The day after, slides were washed several times in PBS-T and PBS-G-T and incubated with Alexa Fluor 488 goat anti-mouse IgG antibody (1:200, Molecular Probes, Eugene, OR, USA, A11029) for 2 h at RT in a humidified chamber in darkness. Sections were washed several times in PBS-T and PBS-G-T in darkness and incubated for 10 min with DAPI at RT, followed by two washes in PBS. Slides were mounted with Mowiol.

### 2.5. TUNEL Technique

The TUNEL technique (Tdt-mediated dUTP Nick End Labeling, Sigma-Aldrich, Madrid, Spain, Cat. No. 11 684 795 910), described by [53], is the histochemical technique commonly used to detect apoptotic nuclei. Cryosections were washed in PBS for 15 min at RT and incubated in 10 µg/mL of proteinase K in PBS for 10 min at 37 °C. The slides were then washed in PBS and incubated in blocking solution (3% H_2_O_2_ in PBS) for 15 min. Subsequently, sections were washed several times in PBS and then incubated for 60 min at 37 °C with TUNEL reaction mixture, consisting of the enzyme terminal deoxynucleotidyl transferase (TdT) and fluorescein-conjugated nucleotides in a reaction buffer. After rinsing in PBS, sections were incubated in blocking solution (PBS-G-T-L) and covered with the HRP-conjugated anti-fluorescein antibody solution. The apoptotic nuclei were visualized using DAB as a chromogen. The sections were then washed thrice in PBS, dehydrated, and mounted with Eukitt^®^ (Kindler, Freiburg, Germany) for observation. In control sections in which the enzyme TdT was absent from the reaction solution, no stained nuclei were observed.

### 2.6. Quantification of TUNEL-Positive Nuclei

Quantification was performed by counting all TUNEL-positive nuclei in micrographs of the central region of the retina. The surface area of the retina in digital microphotographs was measured using the ImageJ free open-source software package (http://rsb.info.nih.gov/ij/ accessed on 28 January 2021). The density profiles were expressed as the mean ± sem of the number of apoptotic nuclei per square millimeter (an/mm^2^). Similar procedures have been described in the literature [23,34,36]. Statistical analyses were performed using Student’s two-tailed *t*-test. Differences between groups were considered as significant (*) when *p* < 0.05 and (**) when *p* < 0.01.

### 2.7. Image Acquisition and Processing

Toluidine blue-stained, TUNEL, and *SA–β–GAL* and immunofluorescence sections were observed with a bright-field and epifluorescence Nikon Eclipse 80i microscope and photographed using an ultra-high definition Nikon DXM1200F digital camera. Images were processed with Adobe Photoshop CS4.

## 3. Results

### 3.1. Programmed Cell Death in the Developing T. guttata Visual System

**Figure 2:**
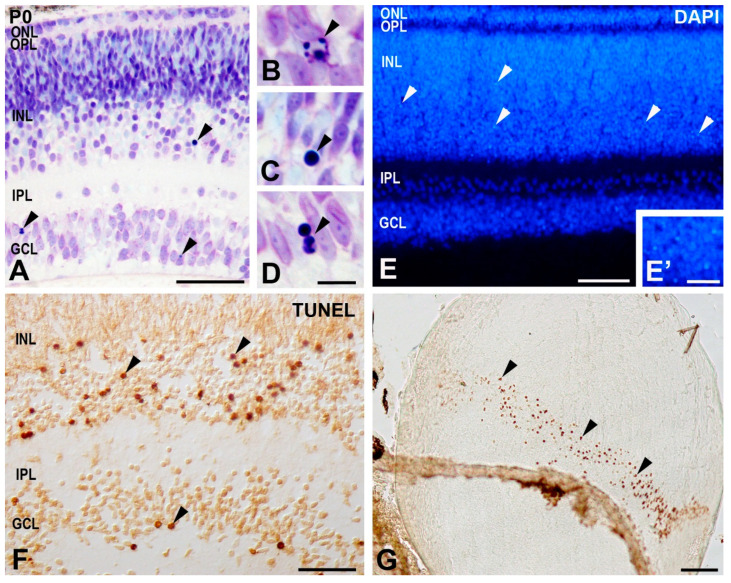
Programmed cell death in the *T. guttata* retina detected by using various sensitive methods. (**A**–**D**) Transversal semi-thin section of the P0 retina showing pyknotic bodies with morphological features typical of apoptosis after toluidine blue staining. (**E**,**E**’) Identification of neuronal cell death in the ganglion cell layer (GCL) and in the inner nuclear layer (INL) (arrowheads) in cryosections of *T. guttata* retinas at P0 stained with DAPI. (**F**,**G**) Eye cryosections of a P0 *T. guttata* hatchling showing intense abundant TUNEL-positive bodies in the GCL and INL (arrowheads in (**C**)) and in the equatorial region of the lens (arrowheads in (**D**)). Abbreviations: GCL, ganglion cell layer; INL, inner nuclear layer; IPL, inner plexiform layer; ONL, outer nuclear layer; OPL, outer plexiform layer. Scale bars: 50 µm (**A**,**E**–**G**), 7 µm (**B**–**D**,**E’**).

In order to identify dying cells in the developing *T. guttata* visual system, we used some of the methods for detecting PCD in embryonic tissues [3]. Light microscopy observation of toluidine blue-stained semi-thin sections revealed pyknotic bodies in the ganglion cell layer (GCL) and in the inner nuclear layer (INL) of the retinal tissue at the hatching day (P0) (Figure 2A–D). Cryosections labeled with DAPI staining identified nuclear condensation in the laminated retina (Figure 2E,E’). Abundant TUNEL-positive nuclei were observed both in the GCL and in the INL (Figure 2F), but also in other eye tissues, such as the lens (Figure 2G) where DNA of cells of the equatorial zone breaks down due to nuclear endodeoxyribonuclease activity [54]. Therefore, PCD was intense and clearly detected in the developing *T. guttata* visual system.

**Figure 3:**
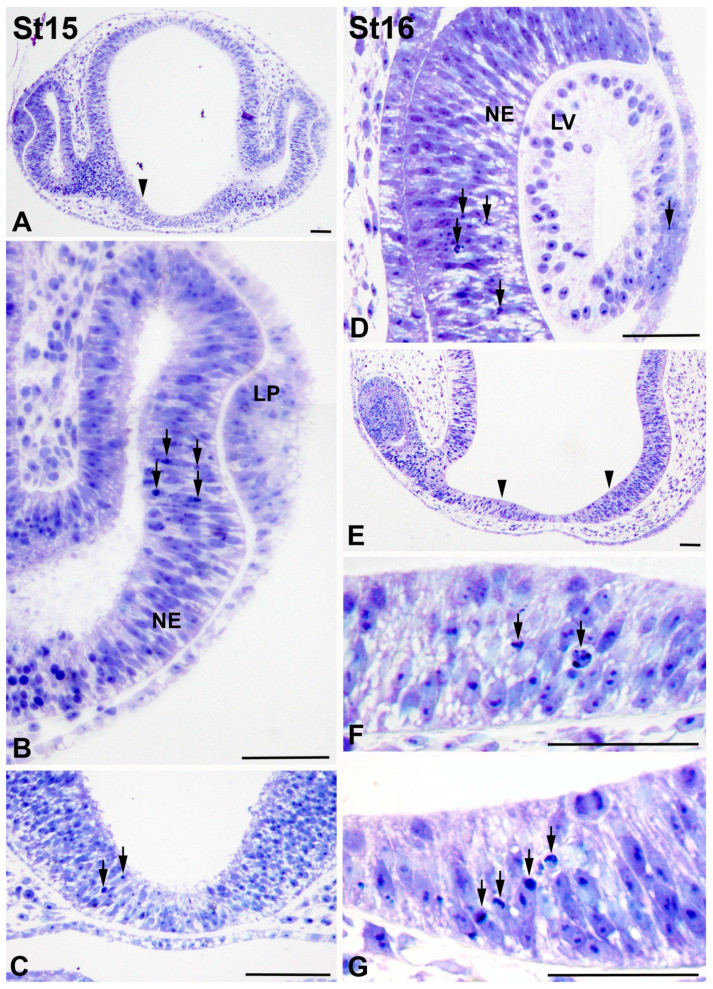
Pyknotic fragments during visual system development in *T. guttata*. Toluidine blue-stained semi-thin sections were obtained from the heads of embryos at different stages of development. Pyknotic bodies were mainly located in the central neural retina ((**A**), arrows in (**B**)) and in the sub-optic necrotic centers (SONCs) (arrowhead in (**A**), arrows in (**C**)) at St15 in the early optic cup. At St16, pyknotic fragments were restricted to the central neural retina, to the anterior wall of the lens vesicle (arrows in (**D**)), and to the SONCs (arrowheads in (**E**), arrows in (**F**,**G**)). Abbreviations: LP, lens placode; LV, lens vesicle; NE, neuroepithelium. Scale bars: 50 µm.

The distribution of pyknotic nuclei and TUNEL-positive bodies was carefully examined from stage 11 (St11), coinciding with the formation of the optic vesicle [48,51], to postnatal day 8 (P8), the last postnatal stage considered in the present study. Pyknotic bodies were absent from the optic anlage from St11 to St14 (not shown). At St15, when the lateral wall of the optic vesicle invaginates to form the optic cup, abundant pyknotic bodies were found in the central undifferentiated neural retina (Figure 3A,B). Moreover, dead cell fragments were observed in two groups of neuroepithelial cells located on either side of the presumptive optic chiasm (Figure 3A,C). Similar areas of cell degeneration have been described in the chicken embryo, the so-called sub-optic necrotic centers (SONCs) [40,41]. The distribution of PCD was similar at St16 in the neuroretina (Figure 3D–G), but the presence of pyknotic bodies in the SONCs (Figure 3E–G) increased notably. Furthermore, pyknotic bodies were also detected in the anterior wall of the lens anlage (Figure 3D).

**Figure 4:**
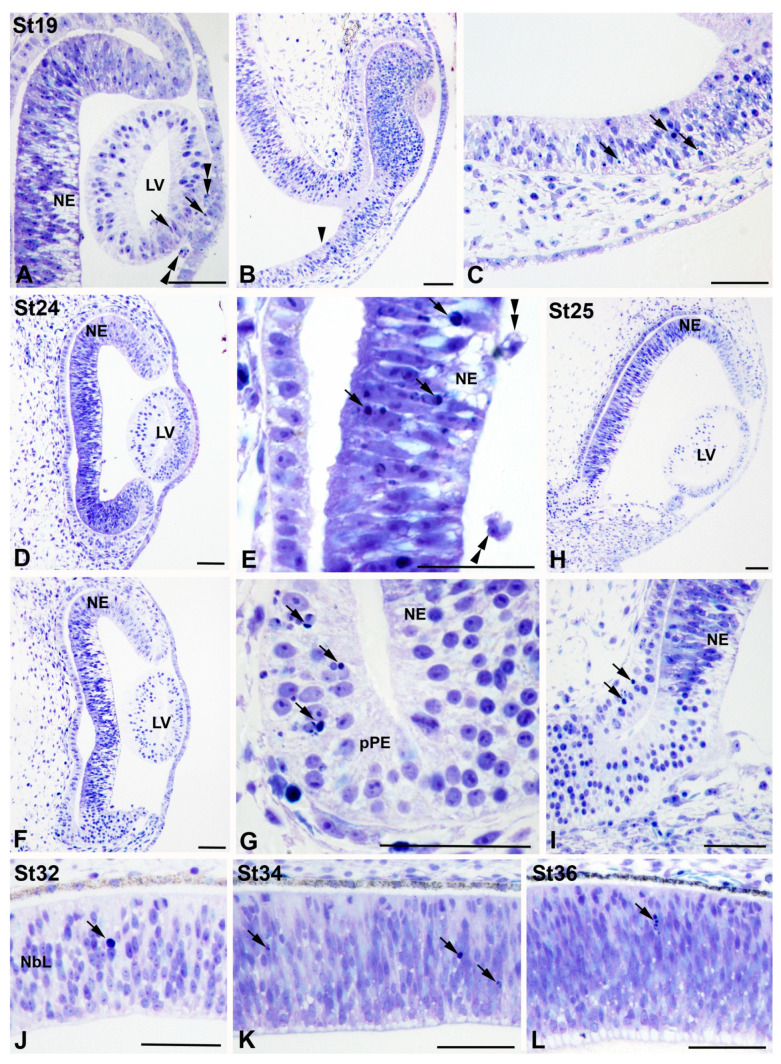
Pyknotic bodies during visual system development in *T. guttata*. Toluidine blue-stained semi-thin sections were obtained from the heads of embryos at different stages of development. At E19, pyknotic fragments were mainly detected in the anterior wall of the lens vesicle (arrows in (**A**)), but also in the SONCs (arrowhead in (**B**), arrows in (**C**)). At St24 (**D**–**G**), pyknotic bodies were concentrated in retinal regions located dorsally to the optic nerve head (arrows in (**E**)) and in the presumptive pigment epithelium located surrounding the optic nerve head (arrows in (**G**)). At St25 (**H**,**I**), abundant pyknotic fragments were detected in the dorsal region of the distal optic nerve (arrows in (**I**)). Pyknotic bodies were sparse and dispersed throughout the neuroblastic layer (NbL) by St32 (arrow in (**J**)), St34 (arrows in (**K**)), and St 36 (arrow in (**L**)). Abbreviations: LV, lens vesicle; NbL, neuroblastic layer; NE, neuroepithelium; pRPE, presumptive retinal pigment epithelium. Scale bars: 50 µm.

At St19, sparse pyknotic bodies were detected in the anterior wall of the lens vesicle (Figure 4A). Pyknotic bodies were still detected in the SONCs (Figure 4B,C). The first differentiating retinal neuroblasts in *T. guttata* appeared by St24 [48,49]. At this stage, pyknotic bodies were concentrated in the NbL in a region located dorsally to the optic nerve head (Figure 4D,E). PCD was also detected in the presumptive retinal pigment epithelium (pRPE), adjacent to the region of the distal optic nerve (Figure 4F,G). At St25, pyknotic bodies were concentrated at the level of the distal optic nerve (Figure 4H,I). From St26 (not shown) to St36, pyknotic bodies were sparsely observed, randomly localized throughout the NbL (Figure 4J–L).

**Figure 5:**
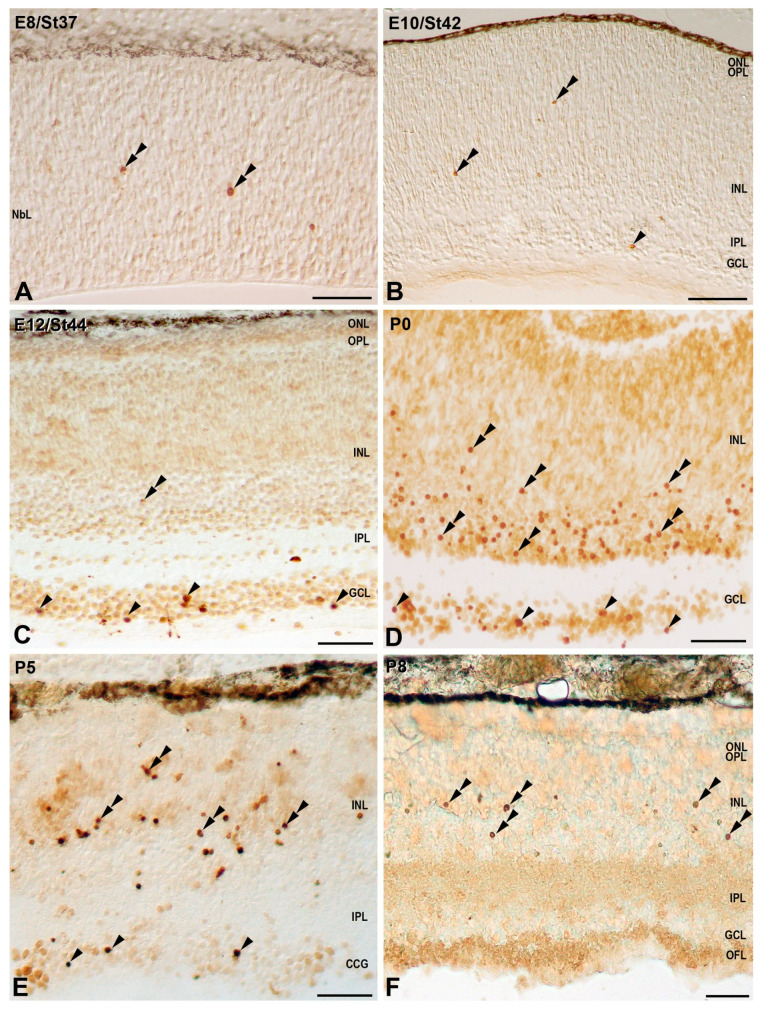
Spatial distribution of TUNEL-positive nuclei in the developing retina of *T. guttata*. Retinal cryosections of embryos and postnatal specimens were treated in accordance with this histochemical technique. Sparse randomly distributed TUNEL-positive nuclei were detected in the NbL at St37 (double arrowheads in (**A**)). At St42, sparse TUNEL-positive nuclei were detected both in the GCL (arrowhead in (**B**)) and in the INL (double arrowheads in (**B**)). TUNEL-positive nuclei were mainly detected in the GCL at St44 (arrowheads in (**B**)), but also in the INL (double arrowhead in (**C**)). TUNEL-positive nuclei progressively diminished from P0 to P8 in the GCL (arrowheads in (**D**,**E**)), but they increased markedly from P0 to P5 in the INL (double arrowheads in (**D**,**E**)). At P8, TUNEL-positive nuclei in the INL were less abundant than observed at previous stages (double arrowheads in (**F**)). Abbreviations: GCL, ganglion cell layer; INL, inner nuclear layer; IPL, inner plexiform layer; NbL, neuroblastic layer; ONL, outer nuclear layer; OPL, outer plexiform layer. Scale bars: 50 µm.

**Figure 6:**
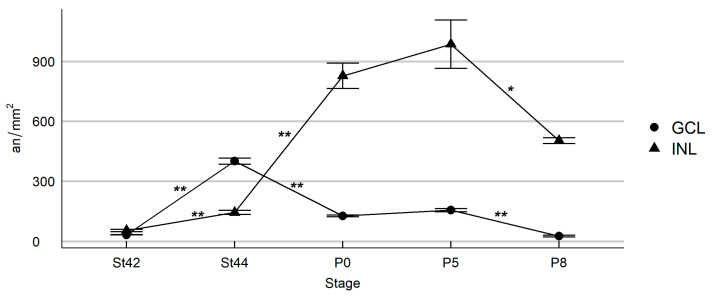
Quantitative analysis of the density of TUNEL-positive nuclei during *T. guttata* retinal histogenesis. The incidence of cell death rose significantly in the GCL between St42 and St44, reaching the highest values in this layer by this latter stage. From P0 onwards, the density of TUNEL-positive nuclei in the GCL diminished progressively. The INL contained a low density of TUNEL-positive nuclei between St42 and St44. A significant increase in the density of TUNEL-positive nuclei was observed at P0, reaching a maximum at P5. At P8, there was a high incidence of cell death in the INL. Abbreviations: an/mm^2^, apoptotic nuclei per square millimeter; GCL, ganglion cell layer; INL, inner nuclear layer. Asterisks correspond to *p* values; * *p* < 0.05 and ** *p* < 0.01.

At St37, scattered TUNEL-positive nuclei were found dispersed throughout the NbL (Figure 5A), similar to the distribution of pyknotic nuclei described from St26 to St36. At St42, retinal stratification was evident, and a few TUNEL-positive nuclei were observed in the GCL and in the INL (Figure 5B and Figure 6). The incidence of cell death rose significantly in the GCL between St42 and St44 (Figure 5C and Figure 6) (2 days before hatching), reaching the highest values in this layer by this stage (Figure 6). At P0, the density of TUNEL-positive nuclei in the GCL diminished (Figure 5D and Figure 6), but increased significantly in the INL, reaching a peak at P5 (Figure 5E and Figure 6). At P8, the last stage analyzed, there was a high incidence of cell death in the INL (Figure 5F and Figure 6), but TUNEL-positive nuclei almost disappeared from the GCL (Figure 5F), reaching values close to 0 in this layer (Figure 6).

**Figure 7:**
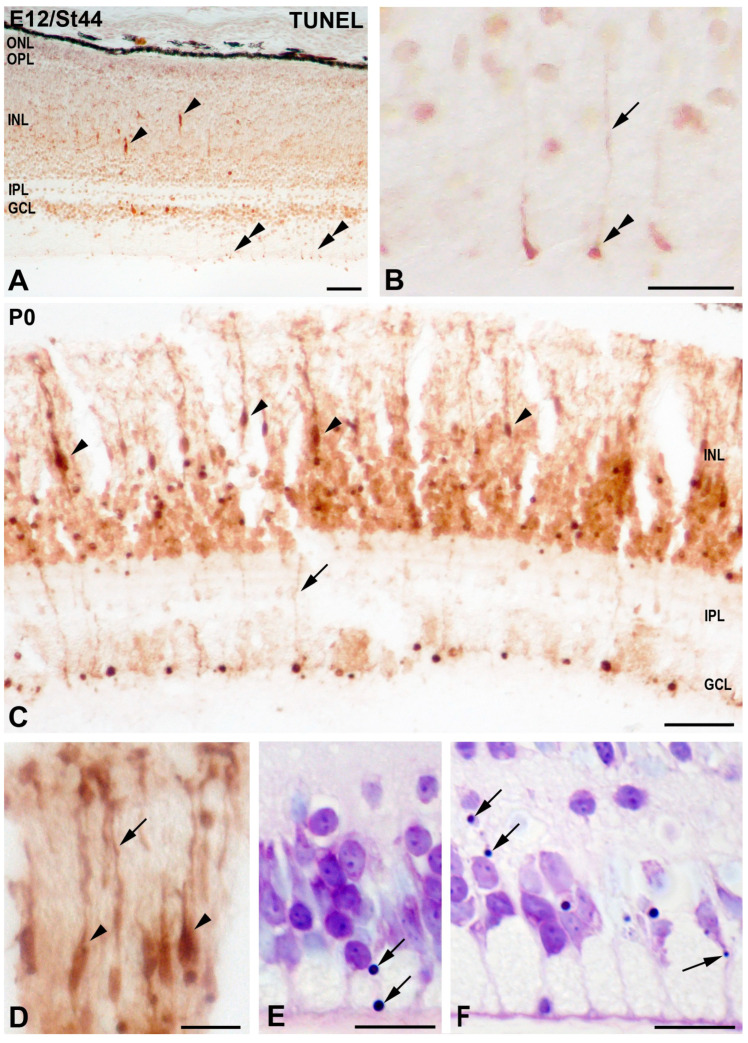
Spatial distribution of TUNEL-positive elements in the developing retina of *T. guttata*. Retinal cryosections of embryos (St44: (**A**,**B**)) and newly hatched chicks (P0: (**C**,**D**)) were treated in accordance with this histochemical technique. Elongated cell somata located in the INL (arrowheads in (**A**,**C**,**D**)) and fine processes (arrows in (**B**–**E**)) of radially oriented cells were diffusely labeled with this technique. Occasionally, TUNEL-positive Müller cell endfeet were labeled in the vitreal surface of the retina (double arrowheads in (**A**,**B**)). Semi-thin sections treated according to the toluidine blue technique revealed small pyknotic bodies within the cytoplasm of Müller cells (arrows in (**E**,**F**)). Abbreviations: GCL, ganglion cell layer; INL, inner nuclear layer; IPL, inner plexiform layer; ONL, outer nuclear layer; OPL, outer plexiform layer. Scale bars: 50 µm in (**A**,**C**); 10 µm in (**B**,**D**–**F**).

At late embryonic stages (St44) (Figure 7A,B) and at P0 (Figure 7C–F), TUNEL-labeling was occasionally detected in the cell somata and in fine processes of radially oriented cells with an apparent intact healthy morphology (Figure 7A–D). Some of the vitreal TUNEL-positive processes form endfeet that seemed to be anchored to the inner limiting membrane ILM (Figure 7A,B). In semi-thin sections, pyknotic bodies were found radially aligned in the cytoplasm of cell processes (Figure 7E,F).

Finally, it is important to note that cell death was completely absent from the ONL during all the embryonic stages and postnatal ages analyzed. Furthermore, the chronotopographical distribution of TUNEL-positive nuclei in the developing *T. guttata* retinal tissue from St42 onwards followed central-to-peripheral and vitreal-to-scleral gradients, in concordance with the gradients of cell differentiation described in this altricial bird species [48,55].

### 3.2. Senescence Markers in the Developing T. guttata Visual System

**Figure 8:**
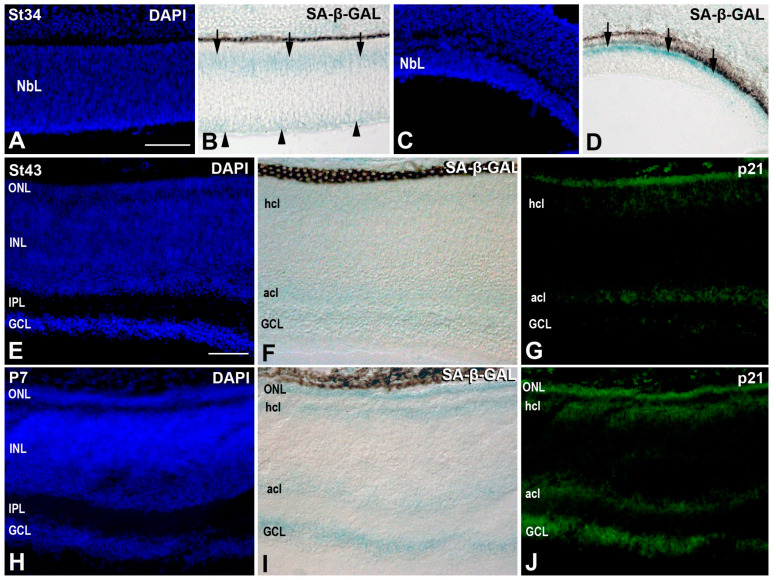
Distribution of SA-*β*-GAL labeling and p21 immunoreactivity in retinal cryosections of embryos (**A**–**G**) and post-hatched specimens (**H**–**J**) of *T. guttata*. All sections were counterstained with DAPI. DAPI staining showed that at St34, the retinal tissue comprised an NbL (**A**,**C**). SA-*β*-GAL activity presented two bands of labeling located in the vitreal and scleral regions of the NbL in the central retina (**B**), but in a single band located sclerally in the peripheral retina (**D**). At St43 and P7, DAPI staining revealed the central retina to present a multi-laminated structure (**E**,**H**). SA-*β*-GAL activity was mainly detected in the GCL, amacrine cell layer, horizontal cell layer, and photoreceptor cell layer (**F**,**I**). The p21 immunosignal was highly coincident with SA-*β*-GAL staining (**G**,**J**). Abbreviations: acl, amacrine cell layer; GCL, ganglion cell layer; hcl, horizontal cell layer; INL, inner nuclear layer; IPL, inner plexiform layer; NbL, neuroblastic layer; ONL, outer nuclear layer; OPL, outer plexiform layer. Scale bars: 50 µm.

Retinal cryosections of zebra finch embryos and hatchlings were stained with SA-*β*-GAL histochemistry and examined for the appearance of positively stained cells. At St34, the vitreal-most region and the scleral surface of the central NbL appeared faintly stained with SA-*β*-GAL histochemistry (Figure 8A,B). In contrast, SA-*β*-GAL staining was mainly detected in the scleral region of the peripheral rim of the retina (Figure 8C,D). The staining pattern of SA-*β*-GAL changed with the appearance of plexiform layers. At St43, SA-*β*-GAL labeling was mainly detected in the GCL, amacrine cell layer, and horizontal cell layer (Figure 8E,F). Double labeling with antibodies against p21 (inhibitor of cyclin-dependent kinases), which has been demonstrated to be overexpressed in senescent cells during embryonic development [1,2,4], showed a strong parallelism between the SA-*β*-GAL signal and p21 immunoreactivity (Figure 8E–G). The same staining patterns were detected in the retina of *T. guttata* hatchlings (Figure 8H–J). 

These staining patterns of cell senescence markers were homogeneous throughout the GCL, amacrine, and horizontal cell layers. Furthermore, TUNEL-positive bodies in the horizontal cell layer were almost absent. Therefore, PCD and senescence markers did not correlate in the developing bird retina.

## 4. Discussion

We have presented details of the distribution of pyknotic bodies and TUNEL-positive nuclei during development of the visual system in the altricial bird species *T. guttata*. Previous work in our laboratory has shown that these are effective methods for the detection of dying cells in the developing visual system of vertebrates (for a review, see [3]). To the best of our knowledge, the present study provides the first description of the spatiotemporal distribution of dying cells in an altricial bird species. Furthermore, in order to find any possible coincidence between apoptotic and senescent cells in the developing visual system, we also labeled retinal cryosections with SA-*β*-GAL histochemistry and p21 immunohistochemistry. All the results will be discussed below.

### 4.1. Cell Death during Early Visual System Morphogenesis in T. guttata

During optic cup stages, abundant pyknotic bodies were found in the central region of the neural retina, coinciding with previous results described in the chicken [32,33,35] and in the mouse [30,31,35]. This wave of PCD may be involved in shaping the optic cup [3]. 

With respect to the *T. guttata* lens vesicle, pyknotic bodies appeared during detachment of this structure from the head ectoderm, coinciding with results described in all vertebrates studied [19,27,31,56,57]. In this case, cell death seems to be involved in eliminating cells in the interface between the ectoderm and lens tissue, facilitating the separation of the lens vesicle.

Finally, abundant pyknotic nuclei were detected in the SONCs, areas of intense cell degeneration located laterally to the ventral midline of the diencephalon in the chicken [40,41] and in the mouse [31]. SONCs were detected between St15 and St20. This wave of cell death preceded the arrival of ganglion cell axons at the presumptive optic chiasm and therefore seems to be involved in the invasion of pioneer axons in this region of the visual system.

### 4.2. Cell Death during the Period of Cell Differentiation in the T. guttata Retina

At St24 (E4.5), coinciding with the appearance of the first differentiated neuroblasts in the *T. guttata* retinal tissue [49], pyknotic nuclei were found in the central retina, dorsally to the optic nerve head. At this stage, cell death affects mainly some proliferating neuroepithelial cells and recent newborn neuroblasts, coinciding with the emergence of the pioneer ganglion cell axons [33,36,37,38]. This wave of cell death (known as “early neural cell death”) could be involved in the creation of extracellular channels that facilitate axonal guidance during early stages of ganglion cell differentiation (for reviews, see [3,58]).

An area of cell death was also detected by St25 in the distal optic nerve, at the junction of this structure with the rudiment of the eye. A similar area of degeneration has been described in the small-spotted catshark, *Scyliorhynus canicula* [19], at stages prior to the invasion of the ganglion cell axons. Neurotrophic cell death affected differentiated neurons in the layered *T. guttata* retina. The emergence of the plexiform layers occurred between St38 (E8.5) and St39 (E9) [48], but the presence of TUNEL-positive bodies was sparse until St42 (E11). At St44 (E13), the incidence of cell death in the GCL increased abruptly, reaching a peak by this stage. In contrast, the maximum of cell death density in the INL was reached at P5, indicating a vitreal-to-scleral progression of cell death, similar to the vitreal-to-scleral wave of cell differentiation described in this bird species [48,49].

**Figure 9:**
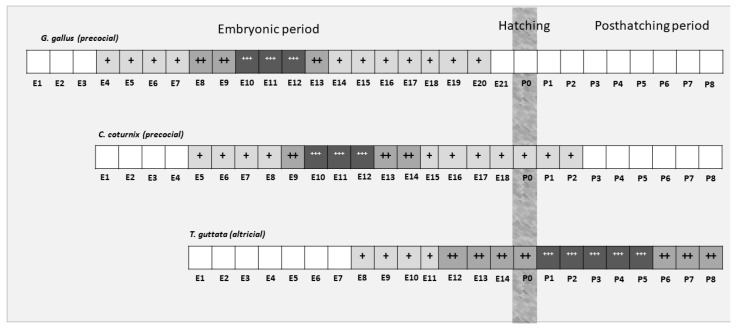
Schematic summary of the chronological patterns and the intensity of neurotrophic cell death in the developing retina of *G. gallus* [34], *C. coturnix* [39], and *T. guttata* (present study). Neurotrophic cell death occurred in the altricial bird at perinatal stages and extended through the first week of life. In contrast, it was restricted to the embryonic period in both of the precocial species. Color codes: white (absence of cell death); light gray (low levels of cell death density) (+); gray (moderate levels of cell death density) (++); dark gray (high levels of cell death density) (+++).

These results also reveal marked differences in the timing of visual system maturation between altricial and precocial bird species (Figure 9). Neurotrophic cell death in the GCL occurs in the quail in the period E8–E14, peaking at E10 [39], while in the chicken, it takes place in the period E8–E15, also peaking at E10 [34]. In contrast, dying ganglion cells are detected in *T. guttata* from embryonic stages (St42–E10.5) to a post-hatching period (P8), peaking at St44 (E12). In the case of the INL, cell death extends from E8 to P1 in the quail, peaking at E12 [39], and from E8 to E19 in the chicken, peaking at E11 [34]. In the present study, we have shown that cell death in the INL is detected from St42 (E10.5) to at least P8, the last stage analyzed in the present study, peaking at P5. Therefore, the highest incidence of cell death in the *T. guttata* INL occurred in the post-hatching period, suggesting that most of the synapses established between retinal cells located in this nuclear layer occur during the first week of life. This is a very interesting finding which suggests that, during early post-hatching life, the retinal tissue is still immature and is unable to process the light information it receives.

Previous studies in our laboratory have shown that mitotic activity is intense during the first postnatal week in the retina of this altricial species [50], reinforcing the idea of the immature state of this tissue during early life. Indeed, *T. guttata* hatchlings open their eyes at P7 [59], coinciding with a decrease in the incidence of cell death in the retina.

These differences in the timing of ontogenetic cell death between altricial and precocial species have been found in all vertebrates studied. The main waves of cell death occur during the embryonic period in precocial fish [16,19], reptiles [23,24,25], and birds [33,34,39,40]. In contrast, cell death takes place mainly after hatching/birth in altricial fish [18], birds (present study), and most of the mammals studied [26,29,31,55].

### 4.3. TUNEL Labeling in the Cytoplasm of Radially Oriented Cells

Diffuse TUNEL-labeling was also found in the cytoplasm of cells that have a bipolar morphology in the radial plane. Their somas were located at the center of the INL, from which radially oriented processes emerge to span the thickness of the neuroretina. Similar results have been described in the developing retina of fish [19], reptiles [23], birds [14], and mammals [60]. Similar staining following retinal injury has also been described in the retina of fish [61,62,63] and mammals [64,65]. These radially oriented TUNEL-positive cells were also GS-immunoreactive [62,63,65,66]. The morphology and immunochemical profiles of these labeled cells coincided with those described for Müller cells [67]. Müller glia possess phagocytic activity to remove degenerating cells during development or under experimental conditions (reviewed in [66]). This cytoplasmic labeling is due to the engulfment of TUNEL-positive cell debris by the phagocytic Müller cells.

### 4.4. Senescence Markers in the Developing Retina of T. guttata

Cellular senescence occurs in different embryonic tissues during restricted time windows, in most cases contributing to degeneration of the interdigital mesoderm [4,6], pronephros [9], mesonephros [1], and developing heart [7] or inner ear [12,13,14] structures. SA-*β*-GAL histochemistry is widely used as a biomarker of cellular senescence in vivo and in vitro [42], even in whole-mount embryos [1,2,4,7,9,11]. Most of these works report that SA-*β*-GAL labeling strongly correlates with areas of cell death. The developing visual system of vertebrates is also affected by several waves of cell death (for a review, see [3]), which we also detected in the *T. guttata* visual system (see above). However, we found no correlation of the labeling pattern of SA-*β*-GAL activity with the TUNEL-positive nuclei detected in the developing retina, in concordance with previous results obtained in the developing chicken retina [43,44]. In this sense, we clearly demonstrated that SA-*β*-GAL activity was restricted to several subpopulations of differentiated neurons (ganglion, amacrine, and horizontal cells) in the embryonic *T. guttata* retina.

Furthermore, the establishment of the state of cell senescence in embryos is associated with the expression of anti-proliferative mediators, such as p21 that seems to act independently of p53 [1,2]. It has been described that p21 expression in mouse embryos strongly correlates with known locations of developmental senescence [68]. In the present study, p21 immunoreactivity faithfully correlates with SA-*β*-GAL labeling, similar to results described in the developing chicken eye [43,44]. Therefore, the present work has clearly shown that the expression of typical senescence markers, including SA-*β*-GAL and p21, in the developing bird retina is up-regulated in subpopulations of differentiated neurons. Notably, both markers have been found to be highly expressed by the first differentiating retinal neurons in the chicken [43,44]. These data indicate that senescence is not the only developmental event that can increase SA-*β*-GAL activity and p21 expression in embryonic tissues. Senescent cells and differentiated retinal neurons share a common biological feature—they are in a characteristic non-proliferative state. Therefore, SA-*β*-GAL activity and p21 could be involved in distinct biological phenomena such as cell senescence and terminal cell differentiation of neurons. In this sense, typical senescence markers have been found to be associated with cell differentiation in the developing tendons [6] and the maturing ventricular myocardium of embryonic mice [7]. However, the possible relationship between the mechanistic events involved in cell senescence and terminal cell differentiation remains to be clarified.

## 5. Conclusions

Relative to precocial bird species, in altricial species, some aspects of brain maturation such as telencephalic neurogenesis are delayed into the post-hatching period [69,70,71,72,73]. Retinal neurogenesis is intense in altricial birds at hatching [48,49] and during the first week of life [50]. Furthermore, it has been demonstrated [74] that the formation of some retinal structures, the foveal pit in particular, is delayed until the second week of life (P10–P14). In the present study, we have demonstrated that there is intense ontogenetic cell death in the retina of the hatched animals. Thus, *T. guttata* constitutes an excellent model in which to study retinal development events during the first weeks of life.

## Figures and Tables

**Table 1:** *T. guttata* embryos and hatchlings used in the present study.

Stage	*n*	Incubation Time (Approximate)
St11	2	54 h
St15	3	66 h
St16	3	3 days
St19	3	3.5 days
St20	3	3.5 days
St24	3	4.5 days
St25	3	5 days
St37	3	8 days
St42	3	11 days
St44	3	13 days
P0	3	14 days
P1	3	15 days
P5	3	19 days
P8	3	22 days

## Data Availability

Some or all data used during the study are available from the corresponding author by request.
